# Qualitative Comparison of LED and LASER Effects on Cutaneous Wound Healing: A Systematic Review of Experimental Studies

**DOI:** 10.1002/cbf.70161

**Published:** 2026-01-15

**Authors:** Mariana Bezerra Miranda, Ana Carolina Silva Barros, Rebeca Barbosa da Rocha, Alessandra Tanuri Magalhães, Vinicius Saura Cardoso

**Affiliations:** ^1^ Postgraduate Program in Biomedical Sciences, Federal University of the Parnaíba Delta (UFDPar) Parnaíba Piauí Brazil; ^2^ Biosignals Laboratory Faculty of Physiotherapy, Federal University of the Parnaíba Delta (UFDPar) Parnaíba Piauí Brazil; ^3^ Postgraduate Program in Biotechnology, Federal University of the Parnaíba Delta (UFDPar) Parnaíba Piauí Brazil; ^4^ Integrated Center for Medical Specialties Parnaíba Piauí Brazil

**Keywords:** animal experimentation, in vitro techniques, LED, LLLT, photobiomodulation, wound healing

## Abstract

This systematic review presents qualitative comparisons between LED and LASER photobiomodulation in cutaneous wound healing, limited by the substantial heterogeneity of experimental parameters. Chronic wounds represent a major clinical challenge due to prolonged healing times, high risk of infection, and increased healthcare costs. Photobiomodulation (PBM) using low‐level laser therapy (LLLT) and light‐emitting diode (LED) devices has emerged as a promising therapeutic strategy for tissue repair. However, the equivalence of the biological and therapeutic effects of these light sources remains debated. The aim of this review is to clarify these effects by systematically comparing LLLT and LED sources on cutaneous wound healing. Comprehensive searches were conducted in PubMed, Web of Science, Scopus, and Embase using terms related to wound healing, LLLT, and LED. We included exclusively controlled in vivo or in vitro studies that directly compared the effects of LLLT and LED on cutaneous wound healing or related biological processes. Of 2020 identified studies, 16 met the inclusion criteria. Both LED and laser promote relevant cellular effects, contributing to the tissue repair process. Findings indicate that both devices promote similar photobiomodulatory effects by activating key cellular and molecular mechanisms essential for wound repair. This review presents predominantly qualitative comparisons between LED and LASER in wound healing, limited by the substantial heterogeneity of experimental parameters. Nevertheless, the available evidence indicates that, although LLLT shows quantitatively superior effects on parameters such as blood vessel density and caliber and type I collagen deposition, both modalities demonstrate similar preclinical effects. (International Prospective Register of Systematic Reviews registration number: CRD420251086145).

## Summary

- Chronic wounds present a major clinical challenge, characterized by delayed healing, heightened risk of infection, and significant healthcare costs.
- Photobiomodulation, using low‐level laser therapy (LLLT) and light‐emitting diodes (LEDs), has been widely investigated as a therapeutic strategy to accelerate repair.
- However, debate continues regarding whether these two light sources produce equivalent biological effects.
- This systematic review compiled in vitro and in vivo studies that directly compared the effects of LED and LLLT on skin wound healing.
- Findings revealed that both modalities enhanced cell viability, proliferation, migration, inflammatory modulation, collagen deposition, and angiogenesis.
- While LLLT demonstrated quantitatively superior outcomes for certain parameters, such as blood vessel density and type I collagen deposition, the overall effects were comparable.
- These results support clinical decision‐making based not only on biological efficacy but also on practical considerations such as cost, availability, and wound characteristics.

## 1 Introduction

A wound is defined as an interruption in the anatomical and functional continuity of tissue, ranging from a simple epithelial breach to deep lesions involving the dermis, subcutaneous tissue, and, in severe cases, muscles, blood vessels, and organs [1]. Wound healing is a complex, dynamic biological process that progresses through sequential and interdependent phases: hemostasis, inflammation, proliferation, and remodeling. Successful repair depends on the orderly and continuous progression of these phases [2, 3]. Failure of this progression results in chronic wounds, which negatively affect quality of life and increase the financial burden on healthcare systems [4, 5].

The economic impact of wound management is substantial, with costs estimated at US$28.1–96.8 billion annually [6]. Loss of the skin barrier further predisposes wounds to bacterial colonization, increasing morbidity, mortality, and treatment costs [7, 8]. Prolonged healing also drives up expenses by increasing the frequency of dressing changes, use of products, and time required from healthcare professionals [9].

Low‐level laser therapy (LLLT) and light‐emitting diode (light‐emitting diode (LED) has emerged as effective strategies for tissue repair [10, 11]. Although both emit light within a narrow spectral range, they differ in beam properties: LLLT is collimated, coherent, and monochromatic, whereas LED light is non‐coherent and less collimated [12]. The divergent nature of LED light limits tissue penetration but allows for more uniform coverage of large, superficial areas [13]. Conversely, LLLT penetrates deeper, effectively stimulating fibroblasts and blood vessels within the dermis [12].

At the cellular level, the therapeutic effects of light result from photobiological reactions triggered when endogenous chromophores such as cytochrome c oxidase (COX) in mitochondria [14]. This initiates a cascade of biochemical events that enhance ATP production, modulate oxidative stress, stimulate collagen synthesis, promote growth factor release, and activate cell signaling pathways, thereby boosting cell proliferation and migration [15, 16].

Despite differences in coherence and penetration, both LLLT and LED have demonstrated therapeutic potential [11, 17]. However, debate persists regarding the equivalence of their biological and clinical effects. This review sought to clarify these effects by systematically comparing coherent (LLLT) and non‐coherent (LED) light sources in skin wound healing. To the best of our knowledge, this is the first systematic review to directly and exclusively compare LLLT and LED in experimental models of skin wound repair, providing an unprecedented synthesis of their relative effects.

## 2 Methods

This systematic review was conducted in accordance with the Preferred Reporting Items for Systematic Reviews and Meta‐Analyses (PRISMA) guidelines [18]. The study protocol was registered with the International Prospective Register of Systematic Reviews (PROSPERO) under the registration number CRD420251086145.

### 2.1 Eligibility Criteria

We included exclusively controlled in vivo or in vitro studies that directly compared the effects of LLLT and LED on cutaneous wound healing or related biological processes, without restriction on the year of publication or language. Studies that combined LED or LLLT with other therapies (pharmacological, invasive, or adjuvant), as well as systematic reviews, narrative reviews, clinical trials, case reports, and observational studies were excluded.

### 2.2 Information Sources and Search Strategy

A comprehensive search was conducted in PubMed, Scopus, Web of Science, and Embase, with the most recent search completed in November 2025. The strategy was based on the predefined PICO (model, intervention/exposure, comparison/control, outcome) framework and incorporated both controlled vocabulary and free‐text terms combined with Boolean operators. Search terms included wound healing, wounds and injuries, ulcers, skin ulcers, skin lesions, tissue repair, excisional wounds, pressure ulcers, diabetic ulcers, skin burns, fibroblasts, keratinocytes, macrophages, light‐emitting diodes, LED, non‐coherent light, NASA light‐emitting diodes, laser therapy, low‐level light therapy, laser biostimulation, low‐level laser irradiation, low‐power laser irradiation, low‐level laser therapy, and coherent light. Additionally, the reference lists of selected studies were manually screened for relevant articles. Detailed search strategies for each database are presented in Table 1.

**Table 1:** Search strategy used in the databases included in the study.

Databases	Search strategy
PUBMED	#1 ((((((((((((((“Wound Healing”[Title/Abstract]) OR (“Wounds and Injuries”[Title/Abstract])) OR (“Ulcer”[Title/Abstract])) OR (“Skin Ulcer”[Title/Abstract])) OR (“Skin lesions”[Title/Abstract])) OR (“Tissue repair”[Title/Abstract])) OR (“Excisional wound”[Title/Abstract])) OR (“pressure ulcer”[Title/Abstract])) OR (“diabetic ulcer”[Title/Abstract])) OR (“Skin burn”[Title/Abstract])) OR (“Fibroblast*”[Title/Abstract])) OR (“Keratinocyte*”[Title/Abstract])) OR (“Macrophage*”[Title/Abstract])) AND #2 (((((“Light Emitting Diode”[Title/Abstract]) OR (“light‐emitting diode”[Title/Abstract])) OR (“LED”[Title/Abstract])) OR (“Non‐ coherent light”[Title/Abstract])) OR (“NASA light‐emitting diode”[Title/Abstract]))) AND #3 (((((((((“LASER”[Title/Abstract]) OR (“LASER Therapy”[Title/Abstract])) OR (“Low‐Level Light Therapy”[Title/Abstract])) OR (“LLLT”[Title/Abstract])) OR (“LASER Biostimulation”[Title/Abstract])) OR (“Low Level Laser Irradiation”[Title/Abstract])) OR (“Low Power Laser Irradiation”[Title/Abstract])) OR (“Low Level Laser Therapy”[Title/Abstract])) OR (“Coherent light[Title/Abstract]))
SCOPUS	#1 (TITLE‐ABS‐KEY (“Wound Healing”) OR TITLE‐ABS‐KEY (“Wounds and Injuries”) OR TITLE‐ABS‐KEY (“Ulcer”) OR TITLE‐ABS‐KEY (“Skin Ulcer”) OR TITLE‐ABS‐KEY (“Skin lesions”) OR TITLE‐ABS‐KEY (“Tissue repair”) OR TITLE‐ABS‐KEY (“Excisional wound”) OR TITLE‐ABS‐KEY (“pressure ulcer”) OR TITLE‐ABS‐KEY (“diabetic ulcer”) OR TITLE‐ABS‐KEY (“Skin burn”) OR TITLE‐ABS‐KEY (“Fibroblast*”) OR TITLE‐ABS‐KEY (“Keratinocyte*”) OR TITLE‐ABS‐KEY (“Macrophage*”)) AND #2 (TITLE‐ABS‐KEY (“Light Emitting Diode”) OR TITLE‐ABS‐KEY (“light‐emitting diode”) OR TITLE‐ABS‐KEY (“LED”) OR TITLE‐ABS‐KEY (“Non‐ coherent light”) OR TITLE‐ABS‐KEY (“NASA light‐emitting diode”)) AND #3 (TITLE‐ABS‐KEY (“LASER”) OR TITLE‐ABS‐KEY (“LASER Therapy”) OR TITLE‐ABS‐KEY (“Low‐Level Light Therapy”) OR TITLE‐ABS‐KEY (“LLLT”) OR TITLE‐ABS‐KEY (“LASER Biostimulation”) OR TITLE‐ABS‐KEY (“Low Level Laser Irradiation”) OR TITLE‐ABS‐KEY (“Low Power Laser Irradiation”) OR TITLE‐ABS‐KEY (“Low Level Laser Therapy”) OR TITLE‐ABS‐KEY (“Coherent light”))
WEB OF SCIENCE	#1 “LASER” (Title) or “LASER Therapy” (Title) or “Low‐Level Light Therapy” (Title) or “LLLT” (Title) or “LASER Biostimulation” (Title) or “Low Level Laser Irradiation” (Title) or “Low Power Laser Irradiation” (Title) or “Low Level Laser Therapy” (Title) or “Coherent light” (Title) AND #2 “Light Emitting Diode” (Title) or “light‐emitting diode” (Title) or “LED” (Title) or “Non‐ coherent light” (Title) or “NASA light‐emitting diode” (Title) AND #3 “Wound Healing” (Title) or “Wounds and Injuries” (Title) or “Ulcer” (Title) or “Skin Ulcer” (Title) or “Skin lesions” (Title) or “Tissue repair” (Title) or “Excisional wound” (Title) or “pressure ulcer” (Title) or “diabetic ulcer” (Title) or “Skin burn” (Title) or “Fibroblast*” (Title) or “Keratinocyte*” (Title) or “Macrophage*” (Title) #1 ‘wound healing’:ti OR (wounds:ti AND injuries:ti) OR ‘ulcer’:ti OR ‘skin ulcer’:ti OR ‘skin lesions’:ti OR ‘tissue repair’:ti OR ‘excisional wound’:ti OR ‘pressure ulcer’:ti OR ‘diabetic ulcer’:ti OR ‘skin burn’:ti OR ‘fibroblast*’:ti OR ‘keratinocyte*’:ti OR ‘macrophage*’:ti AND #2 ‘laser’:ti OR (wounds:ti AND injuries:ti) OR ‘laser therapy’:ti OR ‘low‐level light therapy’:ti OR ‘lllt’:ti OR ‘laser biostimulation’:ti OR ‘low level laser irradiation’:ti OR ‘low power laser irradiation’:ti OR ‘low level laser therapy’:ti OR ‘coherent light’:ti AND #3 ‘light emitting diode’:ti OR ‘light‐emitting diode’:ti OR ‘laser therapy’:ti OR ‘led’:ti OR ‘non‐ coherent light’:ti OR ‘nasa light‐emitting diode’:ti

### 2.3 Study Selection Process

After searching for articles, titles and abstracts were initially identified, followed by a full‐text analysis to determine their final inclusion in the study. Study selection was performed independently by two reviewers, with disagreements resolved through discussion. If consensus could not be reached, a third reviewer was consulted to ensure consistency with the inclusion criteria and study objectives.

### 2.4 Data Extraction

Data extraction was independently performed by two reviewers. Extracted information included study title and year, experimental model, wound type, intervention type and parameters, and reported outcomes. For in vitro studies, data on cell viability, migration, and colony formation were collected. For in vivo studies, data on microscopic, histological, and molecular analyses, inflammatory markers, and oxidative stress were extracted. Discrepancies were reviewed and resolved with input from a third reviewer.

### 2.5 Assessment of Methodological Quality

The risk of bias in animal model intervention studies was assessed using the SYRCLE's Risk of Bias (RoB) tool [19]. This tool consists of ten items that evaluate six types of bias: selection, performance, detection, attrition, reporting, and other potential sources. Each item was classified as “YES” (low risk of bias), “NO” (high risk of bias), or “UNCLEAR” (uncertain risk of bias). For in *vitro* studies, the risk of bias was assessed using the methodology proposed by Kulkarni et al. [20]. This tool comprises eight criteria, each rated as “YES” or “NO.” Studies were then categorized as follows: high risk of bias (more than five “NO” responses), moderate risk (two to five “NO” responses), and low risk (zero or one “NO” response). The application of both tools was carried out independently by two reviewers, and any disagreements were resolved by a third reviewer.

## 3 Results

**Figure 1:**
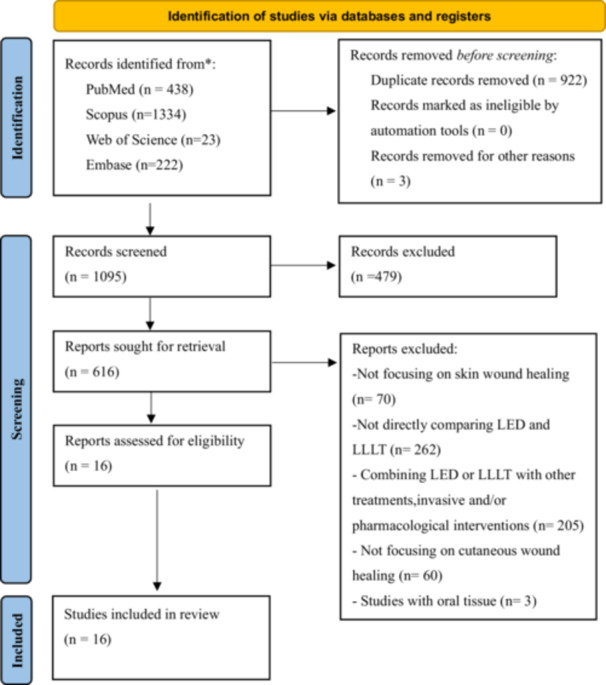
Flowchart of the study selection process according to the Preferred Reporting Items for Systematic Reviews and Meta‐Analyses (PRISMA) guidelines.

The search identified 2020 studies. After removing duplicates and screening titles and abstracts, 616 studies were selected for full‐text review. Sixteen studies published between 2006 and 2024 met the eligibility criteria and were included in the final analysis. The selection process is illustrated in Figure 1.

Among the experimental studies included in the review, four used in vitro designs with cell cultures, whereas 12 employed in vivo preclinical assays using mice, rats, and rabbits. Table 2 summarizes the main characteristics and results of these studies.

**Table 2:** Characteristics and main results of the included studies.

Author/Year	Study model	Caracteristic wound/Assay	Outcome measures	Main results
Ferro et al. 2024 [21]	In vitro: Adipose tissue stem cells (AT)	Scratch assay MTT assay	Viability analysis, in vitro wound healing	LLLT 660 nm (100 mW, 2–4 J) and 830 nm (100 mW, 4 J), as well as LED 630 nm (2 J), promoted increased cell viability when compared to LED 850 nm (60 mW) and the control group. Regarding healing, LLLT 830 nm (40 mW, 4 J) promotes a greater reduction in the lesion area compared to LLLT 660 nm (100 mW, 4 J) and LED 630‐850 nm.
Vinck et al. 2003 [22]	In vitro: Chicken embryo fibroblasts	MTT assay	Cell proliferation analysis	Infrared LED 950 nm and LLLT 830 nm have similar effects on fibroblast proliferation compared to red LED and control. However, green LED 570 nm causes a more significant increase in cell proliferation.
Khan et al. 2016 [23]	In vitro: Epithelial cell, human dermal keratinocytes and normal human oral keratinocytes	Colony formation assay	Colony formation	LLLT 810 nm at 1 and 3 J/cm^2^ was the most effective in stimulating eCFUs compared to LED 660 nm and 850 nm.
Volpato et al. 2011 [24]	In vitro: Balb/c 3T3 fibroblasts (ATCC)	MTT assay	Viability analysis	LLLT 660–780 nm and LED 637 nm promoted an increase in mitochondrial activity and fibroblast viability with no difference between them. Infrared LLLT showed a significant increase in MTT reduction specifically in the 72‐h period in relation to LED 637 nm.
Keshri et al. 2021 [25]	In vivo: mouse 42/ Sprague‐Dawley/ Males/ 180 ± 20 g	Burn, 1.5 cm in diameter	Global quantitative analyses of label‐free proteomics, biochemistry, molecular, ELISA quantification, and IHC.	LED 808 nm and LLLT 810 nm promote similar effects in increasing fibroblast proliferation, angiogenesis, epidermal migration, collagen accumulation, decrease cell apoptosis, increase granulation tissue formation, reduce TNF‐α, IL‐1β, IL‐6 and COX‐2.
Corazza et al. 2007 [26]	In vivo: mouse 120/ Wistar/ Males/ 400–450 g	Excisional, 15 mm de diâmetro	Histological analysis	On Day 14, LED 635 nm (20 J/cm²) and LLLT 660 nm (5 J/cm²) showed the greatest blood vessel formation. On day 21, LLLT 660 nm (5 J/cm²) stood out with the greatest blood vessel density.
Ghaemi et al. 2019 [27]	In vivo: rabbit 20/ Males/ NA	Excisional, 1 cm²	Histopathological and macroscopic analysis	LLLT promotes re‐epithelialization of the epidermis after 30 days, with superior results to irradiation with Blue and Red LED. Between 7 and 10 days, LED and LLLT stimulate the formation of granulation tissue, angiogenesis and increased infiltration of macrophages and lymphocytes.
Tatmatsu‐Rocha et al. 2018 [28]	In vivo: mouse 20/ Wistar/ Males/ 200–250 g	Diabetic wound, excision, 2 cm wide and 2 cm long	Collagen analysis, atomic force microscopy, immunohistochemistry and wound healing	LED 850–680 nm and LLLT 904 nmpromote collagen fiber organization, new blood vessel formation, and increased VEGF. LED demonstrated a more pronounced reduction in COX‐2 expression, higher MFN2 protein expression, and lower FIS1 expression. However, LLLT was more effective in stimulating collagen production.
Silveira et al. 2016 [29]	In vivo: mouse 50/ Wistar/ Males/ 250–300 g	Burn, 20 × 10 mm	Macroscopic analysis of wound contraction, histology, protein analysis by Western Blot, determination of intracellular ROS and nitric oxide, oxidative damage, antioxidant system, and protein content	LED 630 and 850 nm promotes better tissue organization, granulation tissue, hair follicles and well‐developed dermal papillae. LLLT 660 nm showed more developed dermal papillae. LLLT 660 nm and LED 850 nm demonstrated better antioxidant effects.
Oliveira Sampaio et al, 2013 [30]	In vivo: mouse 105/ Wistar/ Males/ ± 50 g	Excisional, 1 cm²	Histological analysis	LLLT 660 nm promotes re‐epithelialization and an increase in the number of fibroblasts and collagen fibers in wounds of non‐anemic animals, while LED 700 nm presents better healing results and a greater number of fibroblasts in anemic animals on Days 7 and 14.
Dall Agnol et al. 2009 [31]	In vivo: mouse 54/ Wistar/ Males/ 180 g e 250 g	Diabetic wound, excision, 0,5 cm^2^	Macroscopic and microscopic analysis	LED 640 nm and LLLT 660 nm similarly reduced inflammatory cells and increased neovascularization and fibroblasts. LED showed faster wound closure at 72 h; however, at 168 h, both promoted a significant reduction in wound diameter compared to the control.
Al‐Watban et al, 2009 [32]	In vivo: mouse 893/ Sprague‐Dawley/ Males/ 408.41 g	Excisional 102,5 mm² or Burn 148 mm²	Reduction of wound area	Both LED 510–872 nm and LLLT 633–980 nm demonstrated positive effects on wound and burn healing in diabetic rats. However, LLLT 633 nm, applied at doses of 5, 10, 20 and 30 J/cm², showed superior results, significantly accelerating healing time in diabetic, non‐diabetic wounds and burns.
De Sousa et al. 2013 [33]	In vivo: mouse 24/ Wistar/ Males/ 200–250 g	Excisional, 1 cm²	Macroscopic analysis, histology, and vessel number counting	LLLT 660–790 nm and LED 530‐700 nm stimulate vascularization and granulation tissue formation, while LED 530 nm promotes more intense re‐epithelialization compared with LLLT and control. Blue LED 460 nm demonstrated intense superficial and dermal vascularization compared with LEDs 530 nm and 700 nm, LLLT 660–790 nm and control.
De Vasconcelos et al, 2015 [34]	In vivo: mouse 100/ Wistar/ Males/ 200–250 g	Burn, 1 cm^2^	Histological, morphological analysis, and quantitative analysis of the collagenization area	LLLT 660–780 nm and LED 520–550 nm have positive effects on the healing of severe burns, however, LLLT 660 nm and 780 nm promote a greater increase in healing in 21 days, less acute inflammation, greater collagen formation and maturation, granulation and re‐epithelialization.
Klebanov et al, 2006 [35]	In vivo: mouse NA/ Wistar/ Males/ 120‐140 g	Excisional, 8–10 mm	Antioxidant activity	At a dose of 0.03 J/cm², both LLLT 632.8 nm and LED 630 nm maintained stable nitrite concentrations. A dose of 1.5 J/cm² stimulated a significant increase in nitrite production on both the 2th and 4th days. In contrast, a higher dose of 4.4 J/cm² led to a marked reduction in nitrite levels.
Klebanov et al. 2006 [36]	In vivo: mouse NA/ Wistar/ Males/	Excisional, 8–10 mm	Phospholipid liposomes	LLLT 632.8 nm and LED 630 nm reduced the levels of TBA‐reactive products and accelerated healing. Irradiation dose of 0.12 J/cm² promotes a slight increase in chemiluminescence.

Abbreviations: ATCC, American type culture collection; cm, centimeters; COX‐2, ciclo‐oxigenase‐2; FIS1, mitochondrial fission protein 1; IL‐1β, interleukin‐1 beta; IL‐6, interleukin‐6; J/cm^2^, joulces square centimeters; LED, light emitting diode; LLLT, low level laser therapy; MFN2, mitofusina‐2; MTT, methylthiazole tetrazolium; NA, not available; nm, nanometers; TBA‐reactive, thiobarbituric acid‐reactive substances; TNF‐α, tumor necrosis factor alpha; VEGF, fator de crescimento endotelial vascular.

### 3.1 Light Characterization

The included studies compared the effects of LED and LLLT with or without sham irradiation or no intervention, with only one study utilizing broadband light [23]. LLLT interventions used wavelengths in the red and infrared ranges, whereas LEDs included red, infrared, green, and blue spectra. Wavelengths ranged from 460 to 950 nm, with 660–830 nm being the most commonly used range [21, 22, 23, 24, 26, 29, 30, 31, 32, 33, 34]. Energy densities ranged from 1 to 60 J/cm², with most studies using 1–10 J/cm². Continuous‐mode irradiation was used in most studies [21, 22, 23, 24, 25, 28, 29, 30, 31, 32, 33], five studies did not specify the mode [26, 27, 34, 35, 36], and only one study directly compared continuous LLLT and pulsed LED [29]. A summary of the irradiation parameters is provided in Table 3.

**Table 3:** Light application parameters.

Author/Year	Group intervention/Comparison	Type de LLLT and LED	Characteristics of the session PBM (wave‐length, dosage, power, application time)	Total intervention time, frequency of sessions per week and/or day	Method and technique of application
Ferro et al. 2024 [21]	Control Group: Sham Intervention: LLLT/LED	LLLT: InGaAlP e GaAlAs	LLLT: 660 nm: dosage: 0,5; 2; 4 J; power: 100 mW and 40 mW; time: 5; 20; 40 s/12,5; 50; 100 s; mode: continuos830 nm: dosage: 0,5; 2; 4 J; power: 40 mW and 50 mW; time: 5; 20; 40/12,5; 50; 100 s; mode: continuosLED: 630 nm: dosage: 0,5; 2; 4 J; power: 50 mW; time: 10; 40; 80 s; mode: continuos850 nm: dosage: 0,5; 2; 4 J; power: 60 mW; time: 8; 32; 64 s; mode: continuos	Once, twice and three times with a 24‐h interval between each application	No contact, 3.3 cm distance
Vinck et al. 2003 [22]	Control Group: Sham Intervention: LLLT/LED	LLLT: GaAlAs	LLLT:830 nm: dosage: 1 J/cm^2^; power: 40 mW; time:5 s; mode: continuosLED:570 nm: dosage: 0,1 J/cm^2^; power: 10 a 0,2 mW; time: 1, 2, 3 min; mode: continuos660 nm: dosage: 0,1 J/cm^2^; power: 80 mW; time: 1, 2, 3 min; mode: continuos950 nm: dosage: 0,1 J/cm^2^; power: 160 mW; time: 1, 2, 3 min; mode: continuos	Daily, for three consecutive days	No contact, 0.6 cm distance
Khan et al. 2016 [23]	Control Group: Sham Intervention: LLLT/LED/Broadband light	NA	LLLT:830 nm and 810 nm: dosage: 1 and 3 J/cm^2^; power: 0,003 e 0,01 W/cm², time: 300 s; mode: continuosLED:660 nm and 850 nm: dosage: 1 and 3 J/cm^2^; power: 0,003 e 0,01 W/cm², time: 300 s; mode: continuosBroadband light:660 nm and 700 nm: dosage: 1 and 3 J/cm^2^; 0,003 e 0,01 W/cm², time: 300 s; mode: continuos	NA	No contact, 14.5 cm distance
Volpato et al. 2011 [24]	Control Group: Sham Intervention: LLLT/LED	LLLT: AlGaInP	LLLT:660 nm: dosage: 4 and 8 J/cm^2^; power: 40 W/cm², time: 4 and 8 s; mode: continuos780 nm: dosage: 5 and 10 J/cm^2^; power: 50 W/cm²; time: 4 and 8 s; mode: continuosLED:637 nm: dosage: 4 and 8 J/cm^2^; power: 40 W/cm², time: 4 and 8 s; mode: continuos	NA	No contact, 1 mm distance
Keshri et al. 2021 [25]	Control Group: Sham Intervention: LLLT/LED	LLLT: GaAlAs	LLLT:810 nm: dosage: 24 J/cm^2^; power: 70 W/cm², time: 10 min; mode: continuosLED:808 nm: dosage: 24 J/cm^2^; power: 70 W/cm², time: 10 min; mode: continuos	7 consecutive days	No contact
Corazza et al. 2007 [26]	Control Group: Sham Intervention: LLLT/LED	NA	LLLT:660 nm: dosage: 5 and 20 J/cm^2^; power: 40 W/cm², time: 221 s and 885 s; mode: NALED:635 nm: dosage: 5 and 20 J/cm^2^; power: 90 W/cm², time: 122 s and 393 s; mode: NA	3 weeks	Contact with the wound surface, six application points on the margin and one in the center of the wound
Ghaemi et al. 2019 [27]	Control Group: Sham Intervention: LLLT/LED	LLLT: AlGalInP	LLLT:650 nm: dosage: 1 J/cm^2^; power: 10 W/cm²; time: 100 s; mode: NALED:630nm‐680nm: dosage: 1 J/cm^2^; power: 75 W/cm²; time: 200 s; mode: NA450nm‐470nm: dosage: 1 J/cm^2^; power: 75 W/cm²; time: 200 s; mode: NA	3 weeks	Contact with the wound surface, six application points on the margin and one in the center of the wound
Tatmatsu‐Rocha et al. 2018 [28]	Control Group: Sham Intervention: LLLT/LED	LLLT: GaAs	LLLT:904 nm: dosage: 18,33 J/cm^2^; power: 40 W/cm²; time: 1 min; mode: continuosLED:850nm‐680nm: dosage: 14,69 J/cm^2^; power: 48 W/cm²; time: 22 s; mode: continuos	Once a day, for 5 days	Contact punctually
Silveira et al. 2016 [29]	Control Group: Sham Intervention: LLLT/LED	LLLT: GaAs and AlGaInP	LLLT:904 nm: dosage: 3 J/cm^2^; power: 70 W/cm²; time: 9 s; mode: continuos660 nm: dosage: 10 J/cm^2^; power: 30 W/cm²; time: 20 s; mode: continuosLED:632 nm: power: 42 W/cm²; time: 10 min; mode: pulsed850 nm: power: 33 W/cm²; time: 10 min; mode: pulsed	7 days, once a day daily	Punctual without contact
Oliveira Sampaio et al. 2013 [30]	Control Group: Sham Intervention: LLLT/LED	NA	LLLT:660 nm: dosage: 10 J/cm^2^; power: 1 W/cm²; time: 260 s; mode: continuosLED:700 nm: dosage: 10 J/cm^2^; power: 0,015 W/cm²; time: 670 s; mode: continuos	48 h intervals for 7, 14 and 21 days	LLLT: four stitches around the wound LED: Single‐point contact over the wound
Dall Agnol et al. 2009 [31]	Control Group: Sham Intervention: LLLT/LED	LED: GaAlAs LLLT: InGaAlP	LLLT:660 nm: dosage: 6 J/cm^2^; power: 30 W/cm²; time: 100 s; mode: continuosLED:640 nm: dosage: 6 J/cm^2^; power: 30 W/cm²; time: 670 s; mode: continuos	An application	No contact, distance 1 cm
Al‐Watban et al. 2009 [32]	Control Group: Sham Intervention: LLLT/LED	NA	LLLT:532, 633, 810, 980 e 10.600 nm: dosage: 5, 10, 20 e 30 J/cm²; power: 140‐143 W/cm²; time:3 min 45 s and 36 min 46 s; mode: continuosLED:510–543, 594–599, 626–639, 640–670 e 842–872 nm: dosage: 5, 10, 20 e 30 J/cm²; power: 200‐300 W/cm²; time: 4 min 5s‐32 min 12 s; mode: continuos	Three times a week	No contact
De Sousa et al. 2013 [33]	Control Group: Sham Intervention: LLLT/LED	NA	LLLT:660 nm: dosage: 10 J/cm^2^; power: 60 W/cm²; time: 168 s; mode: continuos 790 nm: dosage: 10 J/cm^2^; power: 50 W/cm²; time: 200 s; mode: continuos LED:700 nm: dosage: 10 J/cm^2^; power: 15 W/cm²; time: 668 s; mode: continuos 530 nm: dosage: 10 J/cm^2^; power: 8 W/cm²; time: 1250 s; mode: continuos 460 nm: dosage: 10 J/cm^2^; power: 22 W/cm²; time: 456 s; mode: continuos	Alternate days for 7 days	Four stitches around the wound
De Vasconcelos et al. 2015 [34]	Control Group: Sham Intervention: LLLT/LED	LLLT: InGaAIP, AsGaAl, InGaN	LLLT:660 nm: dosage: 10 J/cm^2^; power: 40 W/cm²; time: 10 s; mode: NA780 nm: dosage: 10 J/cm^2^; power: 40 W/cm²; time: 10 s; mode: NALED:520 nm: dosage: 60 J/cm^2^; power: 60 W/cm²; time: 10 s; mode: NA550 nm: dosage: 60 J/cm^2^; power: 60 W/cm²; time: 10 s; mode: NA	NA	No contact, four points
Klebanov et al. 2006 [35]	Control Group: Sham Intervention: LLLT/LED	LLLT: HeNe	LLLT:632,8 nm: dosage: 0,03, 0,9, 1,5, 4,4 J/cm^2^; power: 11 W/cm²; time: NA; mode: NALED:630 nm: dosage: 0,03, 0,9, 1,5, 4,4 J/cm^2^; power: 11 W/cm²; time: NA; mode: NA	Daily for 3 days	No contact, punctually
Klebanov et al. 2006 [36]	Control Group: Sham Intervention: LLLT/LED	LLLT: HeNe	LLLT:632,8 nm: dosage: 0,03, 0,9, 1,5, 4,4 J/cm^2^; power: 11 W/cm²; time: NA; mode: NALED:630 nm: dosage: 0,03, 0,9, 1,5, 4,4 J/cm^2^; power: 11 W/cm²; time: NA; mode: NA	Daily for 3 days	No contact, punctually

Abbreviations: AsGaAl, gallium aluminum arsenate; CG, control group; cm, centimeter; GaAs, aluminum gallium arsenide; HeNe, helium‐neon; InGaAIP, indium gallium aluminum phosphide; InGaN, indium gallium nitride; J/cm^2^, joules square centimeters; LED, light‐emitting diode; LLLT, low‐level laser therapy; mW, megawatt; NA, not available; nm, nanometers.

### 3.2 In Vitro

#### 3.2.1 Cell Viability and Cell Proliferation

Three studies evaluated cell viability using MTT assays [21, 22, 24]. Both red and infrared LED and LLLT promoted increased cell viability and proliferation, with outcomes varying by wavelength, power, and dose [21, 26]. One study demonstrated a superior effect of LED therapy, whereas LLLT produced better outcomes in another study [22, 24]. Ferro et al. [21] reported similar effects with both light modalities.

#### 3.2.2 Cell Migration

Only one study evaluated cell migration using a scratch assay [21]. Both LLLT and LED promoted cell migration, although the effects varied according to wavelength and time point. However, at 6 and 24 h, LLLT produced the greatest reduction in wound area.

#### 3.2.3 Colony Formation Epitelial

One study assessed epithelial colony formation [23]. Khan et al. [23] reported that LLLT 830 and 810 nm and LED 660 and 850 nm all stimulated colony formation. However, lower doses of LLLT at 830 and 810 nm produced a more pronounced increase in both the number and size of epithelial colonies.

### 3.3 In Vivo

#### 3.3.1 Macroscopic Analysis

Six studies performed macroscopic analyses [25, 27, 31, 32, 33, 34]. All studies reported a reduction in wound area following LLLT (633–810 nm) and LED (530–640 nm). Two studies found no significant differences in wound closure between LED and LLLT in diabetic and healthy animals [25, 31]. De Sousa et al. [33] reported enhanced re‐epithelialization with LED at 530 nm compared with other LED wavelengths and LLLT, conversely, one study demonstrated superior effects of LLLT [26]. In animal burn models, LLLT promoted faster wound healing [32, 34].

#### 3.3.2 Microscopic Analysis

Ten studies performed microscopic analyses [26, 27, 28, 29, 30, 31, 32, 34, 35, 36]. Both LLLT (660–810 nm) and LED (635–808 nm) promoted improved cellular organization, enhanced fibroblast proliferation, angiogenesis, collagen accumulation, epidermal migration, and increased expression of Proliferating Cell Nuclear Antigen (PCNA) and Transforming Growth Factor‐β2 (TGF‐β2). Two studies reported reduced neutrophil infiltration and increased lymphocyte and macrophage counts with LED and LLLT [34, 35]. Conversely, LED did not increase collagen fiber content but improved structural organization compared to LLLT [29]. LLLT 660 nm induced better tissue organization and regeneration than LLLT and other LEDs wavelengths [30]. LLLT promoted keratinized epithelium with parallel collagen and spindle‐shaped fibroblasts, reducing inflammation in non‐anemic animals at Day 14, whereas LED produced similar results in anemic animals [31]. LLLT 780 nm yielded greater type I collagen deposition than LED and others LLLT wavelengths [35].

#### 3.3.3 Molecular Analysis

Four studies assessed protein expression [26, 27, 29, 34]. LLLT (660–904 nm) and LED (460–850 nm) modulated proteins involved in proliferation, collagen synthesis, migration, VEGF formation, and extracellular matrix regeneration [27, 29, 34]. One study reported increased DNA, total protein, hydroxyproline, and hyaluronic acid levels with LLLT and LED in burn models compared with controls [27]. Pulsed LLLT regulated 727 differentially expressed proteins linked to cell signaling and neuronal function, while LED modulated 822 proteins, particularly in metabolic and inflammatory pathways [24]. LED was associated with increased mitofusin‐2 (MFN2) and decreased mitochondrial fission protein 1 (FIS1), favoring mitochondrial balance, whereas LLLT increased both proteins, suggesting mitochondrial stress [28].

### 3.4 Expression of Inflammatory Activity Markers

Four studies analyzed inflammatory markers [26, 28, 29, 36]. LLLT and LED reduced TNF‐α, IL‐1β, IL‐6, COX‐2 levels, increased ERK1/2 phosphorylation and produced the highest nitrite levels [26, 29, 36]. LED produced greater COX‐2 reduction than LLLT [28].

### 3.5 Determinants of Oxidative Stress

Three studies evaluated oxidative stress markers [30, 36, 37]. LLLT 904 nm and LED 632 nm increased oxidative stress, whereas LLLT 660 nm and LED 850 nm exerted antioxidant effects, reducing glutathione peroxidase activity and lowering nitrite and carbonyl levels [30]. LLLT and LED at low doses stimulated nitrite production, leukocyte activity and reactive oxygen species generation, whereas higher doses inhibited these processes [36, 37]. Both LED and LLLT reduced lipid peroxidation from day 4 onward compared with controls [37].

### 3.6 Quality Assessment of the Studies

Four studies used an in vitro methodology, three of which were classified as having a moderate risk of bias [21, 22, 24] and one as low risk [23]. The criteria that received the most negative evaluations were related to reporting of cell passage number, missed wells, cell handling between establishment and measurement, and cell characterization. Table 4 summarizes the risk of bias assessment for the in vitro studies.

**Table 4:** Summary of risk of bias of in vitro studies.

Study	Items								
	**1**	**2**	**3**	**4**	**5**	**6**	**7**	**8**	**Result**
Ferro et al. [21]	Yes	Yes	No	Yes	Yes	No	No	Yes	Moderate
Vink et al. [22]	Yes	Yes	Yes	PA	Yes	No	Yes	Yes	Moderate
Khan et al. [23]	Yes	Yes	Yes	PA	PA	No	No	Yes	Low
Volpato et al. [24]	Yes	Yes	Yes	Yes	Yes	No	No	Yes	Moderate

*Note:* 1. Cell type. 2. Were the laser parameters reported for all the study groups? 3. Have cell passages been performed? 4. Cell characterization was performed as previously described. 5. Was the cell culture method used? 6. Was the number of lost wells accurately reported? 7. Was cell culture handling reported between the establishment and measurement? 8. Have all results been reported. Abbreviations: NL, Nothing lost; PA, Partial.

Twelve studies conducted in vivo assays [25, 26, 27, 28, 29, 30]. In all evaluated articles, six of the ten domains of the scale were classified as uncertain risk or high risk. Only three studies explicitly reported random sequence generation of animals [31, 33, 34]. The domain blinded animal intervention was assessed as high risk in all studies. The domains allocation concealment, random housing, random outcome assessment, and other types of bias were rated as uncertain risk in all twelve included studies. The results of the risk of bias (RoB) assessment are presented in Table 5.

**Table 5:** RoB bias risk assessment (Syrcle's RoB tool).

Study	Syrcle Item									
	D1	D2	D3	D4	D5	D6	D7	D8	D9	D10
Keshri et al. 2021 [25]	UR	No	UR	UR	Yes	UR	No	No	No	UR
Corazza et al. 2007 [26]	UR	No	UR	UR	Yes	UR	UR	No	No	UR
Ghaemi et al. 2019 [27]	UR	No	UR	UR	Yes	UR	Yes	No	No	UR
Tatmatsu‐Rocha et al. 2018 [28]	UR	No	UR	UR	Yes	UR	Yes	No	No	UR
Silveira et al. 2016 [29]	UR	No	UR	UR	Yes	UR	No	No	No	UR
Oliveira Sampaio et al. 2013 [30]	UR	No	UR	UR	Yes	UR	No	No	No	UR
Dall Agnol et al. 2009 [31]	No	No	UR	UR	Yes	UR	No	No	No	UR
Al‐Watban et al. 2009 [32]	UR	No	UR	UR	Yes	UR	UR	No	No	UR
De Sousa et al. 2013 [33]	No	No	UR	UR	Yes	UR	No	No	No	UR
De Vasconcelos et al. 2015 [34]	No	No	UR	UR	Yes	UR	No	No	No	UR
Klebanov et al. 2006 [35]	UR	No	UR	UR	Yes	UR	No	No	No	UR
Klebanov et al. 2006 [36]	UR	No	UR	UR	Yes	UR	No	No	No	UR

*Note:* D1: sequence generation. D2: baseline characteristics. D3: allocation concealment. D4: random housing. D5: blinded animal intervention. D6: random outcome assessment. D7: blinding outcome assessors. D8: incomplete outcome data. D9: selective outcome reporting. D10: other types of bias. Abbreviations: No, low risk of bias; UR, uncertain risk; Yes, high risk of.

## 4 Discussion

**Figure 2:**
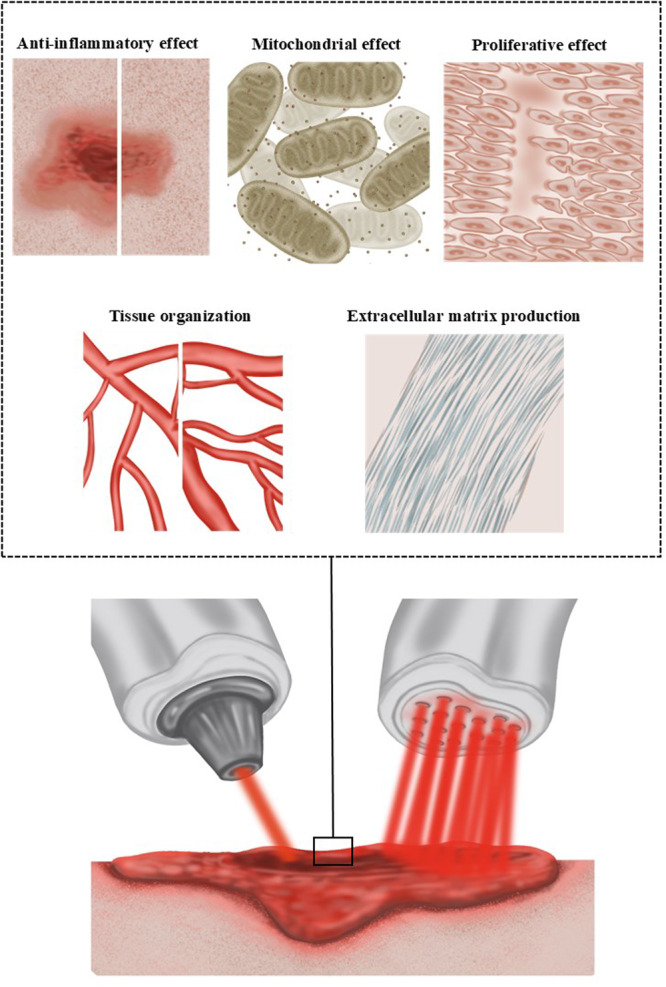
Cellular effects of photobiomodulation with LED and LASER.

This review aimed to compare the healing effects of coherent and non‐coherent light. The studies analyzed demonstrate that both LLLT and LED promote essential cellular and molecular effects in in vivo and in vitro wound‐healing models when compared with control groups. Both light sources effectively modulate fundamental aspects of tissue repair, including cell viability, proliferation, migration, extracellular matrix deposition, and angiogenesis, leading to accelerated wound healing (Figure 2). It should be noted, however, that such comparisons are qualitative and influenced by the substantial heterogeneity of the experimental parameters.

Despite the comparable biological effects observed between LLLT and LED, practical considerations such as cost and accessibility must also be taken into account. LLLT systems are substantially more expensive than LED devices, which may limit their use in clinical applications. On the other hand, LEDs offer a more affordable and safer alternative, justifying their wider adoption in clinical settings [37].

The primary physical characteristics of LLLT devices are the collimation and coherence of their emitted light beams. These properties contrast with those of LED devices, which have lower beam coherence [37]. However, this difference in light‐emission characteristics does not seem to significantly influence the therapeutic outcome, as the results consistently show that both devices can produce similar photobiomodulatory effects at the cellular level. These effects are largely attributed to the photochemical mechanisms of photobiomodulation, which are independent of light coherence [38].

The biological response to photobiomodulation is directly related to the dose‐response relationship and the depth of light penetration into tissues. According to the principle of biphasic response, low doses tend to stimulate cellular activity, while high doses can inhibit proliferation or induce photothermal stress [39]. Therefore, the precise definition of irradiation parameters, including wavelength, fluence, and exposure time, becomes fundamental to ensure consistent therapeutic effects.

Wavelength is crucial for both absorption by chromophores and the depth of tissue action. Red light (600–700 nm) exhibits strong absorption by cytochrome c oxidase and mitochondrial flavoproteins, favoring ATP synthesis and cell proliferation in epidermal and superficial dermal layers. Near‐infrared light (780–950 nm) has greater penetration capacity (5–10 mm), reaching fibroblasts, endothelial cells, and vascular structures located in the dermis and hypodermis [40, 41]. In contrast, blue and green wavelengths (450–550 nm) have limited penetration (< 1 mm), acting mainly in the modulation of bacterial load and superficial wound inflammation [42].

Although both light sources stimulate similar biological mechanisms, several authors suggest that LLLT may have a superior effect in certain aspects. Specifically, LLLT was found to increase blood vessel density and caliber, as well as type I collagen deposition, to a greater extent than LED [26, 34]. Nevertheless, these differences were primarily quantitative, since LED also promoted significant improvements in these parameters compared with controls. This enhanced vascular response may explain the faster lesion closure observed in wounds treated with LLLT. Vascular restoration is a critical step in the healing process, as neovascularization at the injury site is essential for restoring blood flow and ensuring the delivery of oxygen and nutrients required by repair‐associated cells [43].

LLLT also modulates the early inflammatory response by promoting cell recruitment, vasodilation, and local angiogenesis [44]. Additional studies support the role of LLLT in vascularization and collagen deposition. Cury et al. [45] reported that LLLT irradiation (660 and 780 nm; 30 and 40 J/cm²) in ischemic skin flaps enhanced new vessel formation, increased expression of hypoxia‐inducible factor 1‐alpha (HIF‐1α) and VEGF, and reduced MMP‐2 activity. Similarly, in fibroblast cultures, LLLT (904 nm; 2 J/cm²) activated pathways involved in collagen deposition (COL1α1) and VEGF expression, reinforcing its contribution to extracellular matrix synthesis and angiogenesis [46].

Molecular analyses revealed that both devices activate similar pathways, however, LED primarily modulates inflammatory pathways, whereas LLLT predominantly influences proteins associated with cell proliferation [25, 28]. LED activates pathways such as SPHK1/NF‐κB, reducing pro‐inflammatory cytokines (TNF‐α, IL‐1β, COX‐2) and increasing anti‐inflammatory mediators [47, 48]. In contrast, LLLT primarily activates proliferative pathways, including PI3K/Akt and MAPK/ERK, which promote collagen synthesis, angiogenesis, and cell migration, key processes in the later phases of tissue repair [49, 50].

Findings from experimental studies are supported by clinical trials demonstrating that both LED and LLLT accelerate tissue repair and wound healing. Borges et al. [51] treated diabetic foot ulcers (DFUs) with 620 and 904 nm LEDs daily for 12 weeks, reporting complete healing and significant area reduction at both wavelengths. Similarly, Cardoso et al. [52] evaluated three doses of 904 nm LLLT (4, 8, and 10 J/cm²) for DFU treatment and found improved healing rates at all doses, with 10 J/cm² achieving the highest proportion of completely healed ulcers.

This systematic review suggests that the two different types of light coherence used in photobiomodulation produce similar effects in stimulating key cellular events involved in tissue repair. In this context, the energy dose applied may have a greater influence on the outcomes. However, the included studies reported results obtained using devices with varying physical characteristics, even within the same experiment. One of the parameters showing the greatest variability was power, which represents the rate of energy emission over time [53]. Therefore, for future research, we recommend the standardization of irradiation parameters, as the use of standardized reporting templates could enhance data comparability and help clarify whether parameters such as power influence the measured outcomes.

The combination of parameters is also important in determining the temperature variation induced by PBM. In addition to the non‐thermal effects resulting from chromophore stimulation, the absorption of irradiation by the target tissue may lead to an increase in temperature and consequently generate thermal effects. Cronshaw et al. [54] identified temperature variations ranging from 2°C to 12°C, depending on the tissue thickness used in the *in vitro* experiments, beam size, wavelength, irradiance, fluence, and application technique. The temperature rise may activate cellular stimulation mechanisms, however, at higher levels, it can result in cellular inhibition or photothermal damage [54]. The studies included in this review did not compare outcomes from the perspective of thermal effects; therefore, it is suggested that future investigations address this aspect.

Chronic wounds represent a major clinical challenge due to delayed healing, susceptibility to infections, and substantial functional and economic burdens [55]. Although this review presents consistent preclinical evidence, the relevance of these cellular responses to the context of chronic wounds is still uncertain, given that these models exhibit persistent inflammation, hypoxia, phenotypic alterations of fibroblasts, and vascular compromise. Therefore, the next translational step should focus on rigorously characterized chronic animal models capable of reproducing this pathophysiological complexity.

### 4.1 Study Limitations

This review presents important methodological limitations. The main one is the impossibility of normalizing power density between LED and LASER devices, due to the absence, in most studies, of essential information for calculating optical dose (power in the target plane, beam area and profile, and pulse parameters). This gap prevents the standardization of energy between modalities and limits more robust quantitative comparisons. There was also great heterogeneity in irradiation parameters and outcomes evaluated, reducing the internal consistency of the evidence. Furthermore, although the protocol foresaw the inclusion of studies with control, placebo, or sham groups, this review focused only on direct comparisons between LASER and LED.

## 5 Conclusion

In general, evidence indicates that both LED therapy and low‐level laser therapy (LLLT) stimulate cellular and molecular mechanisms essential for tissue repair. Although LLLT demonstrates quantitatively greater effects on parameters such as blood vessel density and caliber and type I collagen deposition, both modalities show similar preclinical effects.

## Ethics Statement

International Prospective Register of Systematic Review Registration approved this study number: CRD420251086145.

## Conflicts of Interest

The authors declare no conflicts of interest.

## Data Availability

The data that support the findings of this study are available from the corresponding author upon reasonable request.

## References

[1] K. M. Vannella and T. A. Wynn , “Mechanisms of Organ Injury and Repair by Macrophages,” Annual Review of Physiology 79 (2017): 593–617, 10.1146/annurev-physiol-022516-034356. 27959618

[2] P. H. Wang , B. S. Huang , H. C. Horng , C. C. Yeh , and Y. J. Chen , Wound Healing, 10.1016/j.jcma.2017.11.002.

[3] S. Guo and L. A. Dipietro , “Factors Affecting Wound Healing,” Journal of Dental Research 89, no. 3 (2010): 219–229, 10.1177/0022034509359125. 20139336 PMC2903966

[4] C. K. Sen , “Human Wound and Its Burden: Updated 2020 Compendium of Estimates,” Advances in Wound Care 10, no. 5 (2021): 281–292, 10.1089/wound.2021.0026. 33733885 PMC8024242

[5] M. Olsson , K. Järbrink , U. Divakar , et al., “The Humanistic and Economic Burden of Chronic Wounds: A Systematic Review,” Wound Repair and Regeneration 27, no. 1 (2019): 114–125, 10.1111/wrr.12683. 30362646

[6] S. R. Nussbaum , M. J. Carter , C. E. Fife , et al., “An Economic Evaluation of the Impact, Cost, and Medicare Policy Implications of Chronic Nonhealing Wounds,” Value in Health 21, no. 1 (2018): 27–32, 10.1016/j.jval.2017.07.007. 29304937

[7] S. L. Percival , K. E. Hill , D. W. Williams , S. J. Hooper , D. W. Thomas , and J. W. Costerton , “A Review of the Scientific Evidence for Biofilms in Wounds,” Wound Repair and Regeneration 20, no. 5 (2012): 647–657, 10.1111/j.1524-475X.2012.00836.x. 22985037

[8] J. Salenius , M. Suntila , T. Ahti , et al., “Long‐Term Mortality Among Patients With Chronic Ulcers,” Acta Dermato Venereologica 101, no. 5 (2021): adv00455, 10.2340/00015555-3803. 33856039 PMC9367040

[9] J. F. Guest , G. W. Fuller , and P. Vowden , “Diabetic Foot Ulcer Management in Clinical Practice in the UK: Costs and Outcomes,” International Wound Journal 15, no. 1 (2018): 43–52, 10.1111/iwj.12816. 29243399 PMC7950039

[10] H. A. Elessawy , W. H. Borhan , N. A. Ghozlan , and S. H. Nagib , “Effect of Light‐Emitting Diode Irradiation on Chronic Nonhealed Wound After Below‐Knee Amputation,” International Journal of Lower Extremity Wounds 20, no. 3 (2021): 251–256, 10.1177/1534734620915108. 32308074

[11] M. B. Miranda , R. F. Alves , R. B. da Rocha , and V. S. Cardoso , “Effects and Parameterization of Low‐Level Laser Therapy in Diabetic Ulcers: An Umbrella Review of Systematic Reviews and Meta‐Umbrella,” Lasers in Medical Science 40, no. 1 (2025): 109. 39982518 10.1007/s10103-025-04366-2

[12] J. Garcia‐Sucerquia , “Color Digital Lensless Holographic Microscopy: Laser Versus LED Illumination,” Applied Optics 55, no. 24 (2016): 6649–6655, 10.1364/AO.55.006649. 27556985

[13] D. R. Opel , E. Hagstrom , A. K. Pace , et al., “Light‐Emitting Diodes: A Brief Review and Clinical Experience,” Journal of Clinical and Aesthetic Dermatology 8, no. 6 (2015): 36–44. PMC4479368 26155326

[14] T. Karu , “Primary and Secondary Mechanisms of Action of Visible to Near‐Ir Radiation on Cells,” Journal of Photochemistry and Photobiology B: Biology 49, no. 1 (1999): 1–17, 10.1016/S1011-1344(98)00219-X. 10365442

[15] T. R. Schmidt , B. C. Mármora , F. T. Brochado , et al., “Red Light‐Emitting Diode on Skin Healing: An In Vitro and In Vivo Experimental Study,” Anais Brasileiros de Dermatologia 100, no. 1 (2025): 54–62, 10.1016/j.abd.2024.02.008. 39521711 PMC11745292

[16] E. Mathioudaki , M. Rallis , K. Politopoulos , and E. Alexandratou , “Photobiomodulation and Wound Healing: Low‐Level Laser Therapy at 661 nm in a Scratch Assay Keratinocyte Model,” Annals of Biomedical Engineering 52, no. 2 (2024): 376–385, 10.1007/s10439-023-03384-x. 37851144 PMC10808316

[17] L. T. N. Ngoc , J. Y. Moon , and Y. C. Lee , “Utilization of Light‐Emitting Diodes for Skin Therapy: Systematic Review and Meta‐Analysis,” Photodermatology, Photoimmunology & Photomedicine 39, no. 4 (2023): 303–317, 10.1111/phpp.12841. 36310510

[18] M. J. Page , J. E. McKenzie , P. M. Bossuyt , et al., “The PRISMA 2020 Statement: An Updated Guideline for Reporting Systematic Reviews,” BMJ 372 (2021): n71, 10.1136/bmj.n71. 33782057 PMC8005924

[19] C. R. Hooijmans , M. M. Rovers , R. B. de Vries , M. Leenaars , M. Ritskes‐Hoitinga , and M. W. Langendam , “SYRCLE's Risk of Bias Tool for Animal Studies,” BMC Medical Research Methodology 14 (2014): 43, 10.1186/1471-2288-14-43. 24667063 PMC4230647

[20] S. Kulkarni , M. Meer , and R. George , “The Effect of Photobiomodulation on Human Dental Pulp‐Derived Stem Cells: Systematic Review,” Lasers in Medical Science 35, no. 9 (2020): 1889–1897, 10.1007/s10103-020-03071-6. 32572661

[21] A. P. Ferro , R. R. de Jesus Guirro , M. D. Orellana , G. C. de Santis , J. A. Farina Junior , and E. C. de Oliveira Guirro , “Photobiomodulation With Laser and Led on Mesenchymal Stem Cells Viability and Wound Closure In Vitro,” Lasers in Medical Science 39, no. 1 (2024): 205, 10.1007/s10103-024-04159-z. 39088075

[22] E. M. Vinck , B. J. Cagnie , M. J. Cornelissen , H. A. Declercq , and D. C. Cambier , “Increased Fibroblast Proliferation Induced by Light Emitting Diode and Low Power Laser Irradiation,” Lasers in Medical Science 18, no. 2 (2003): 95–99, 10.1007/s10103-003-0262-x. 12928819

[23] I. Khan and P. R. Arany , “Photobiomodulation Therapy Promotes Expansion of Epithelial Colony Forming Units,” Photomedicine and Laser Surgery 34, no. 11 (2016): 550–555, 10.1089/pho.2015.4054. 27841965 PMC5116699

[24] L. E. R. Volpato , R. C. de Oliveira , M. M. Espinosa , V. S. Bagnato , and M. A. A. M. Machado , “Viability of Fibroblasts Cultured Under Nutritional Stress Irradiated With Red Laser, Infrared Laser, and Red Light‐Emitting Diode,” Journal of Biomedical Optics 16, no. 7 (2011): 075004, 10.1117/1.3602850. 21806261

[25] G. K. Keshri , G. Kumar , M. Sharma , K. Bora , B. Kumar , and A. Gupta , “Photobiomodulation Effects of Pulsed‐Nir Laser (810 nm) and LED (808 ± 3 nm) With Identical Treatment Regimen on Burn Wound Healing: A Quantitative Label‐Free Global Proteomic Approach,” Journal of Photochemistry and Photobiology 6 (2021): 100024.

[26] A. V. Corazza , J. Jorge , C. Kurachi , and V. S. Bagnato , “Photobiomodulation on the Angiogenesis of Skin Wounds in Rats Using Different Light Sources,” Photomedicine and Laser Surgery 25, no. 2 (2007): 102–106, 10.1089/pho.2006.2011. 17508845

[27] M. Ghaemi , D. Sharifi , S. Mokmeli , G. Kowsari , P. Mortazavi , and P. Golmai , “Comparison and Evaluation of the Low‐Level Laser and the Red and Blue LED Effects on Wound Healing in Rabbit,” Journal of Lasers in Medical Sciences 10, no. 3 (2019): 189–193, 10.15171/jlms.2019.30. 31749944 PMC6817807

[28] J. C. Tatmatsu‐Rocha , C. R. Tim , L. Avo , et al., “Mitochondrial Dynamics (Fission and Fusion) and Collagen Production in a Rat Model of Diabetic Wound Healing Treated by Photobiomodulation: Comparison of 904 nm Laser and 850 nm Light‐Emitting Diode (LED),” Journal of Photochemistry and Photobiology, B: Biology 187 (2018): 41–47, 10.1016/j.jphotobiol.2018.07.032. 30098521 PMC6131055

[29] P. C. L. Silveira , K. B. Ferreira , F. R. da Rocha , et al., “Effect of Low‐Power Laser (Lpl) and Light‐Emitting Diode (LED) on Inflammatory Response in Burn Wound Healing,” Inflammation 39, no. 4 (2016): 1395–1404, 10.1007/s10753-016-0371-x. 27206919

[30] S. C. P. Oliveira Sampaio , J. S. de C Monteiro , M. C. T. Cangussú , et al., “Effect of Laser and Led Phototherapies on the Healing of Cutaneous Wound on Healthy and Iron‐Deficient Wistar Rats and Their Impact on Fibroblastic Activity During Wound Healing,” Lasers in Medical Science 28, no. 3 (2013): 799–806, 10.1007/s10103-012-1161-9. 22814898

[31] M. A. Dall Agnol , R. A. Nicolau , C. J. de Lima , and E. Munin , “Comparative Analysis of Coherent Light Action (Laser) Versus Non‐Coherent Light (Light‐Emitting Diode) for Tissue Repair in Diabetic Rats,” Lasers in Medical Science 24, no. 6 (2009): 909–916, 10.1007/s10103-009-0648-5. 19238507

[32] F. A. H. Al‐Watban , “Laser Therapy Converts Diabetic Wound Healing to Normal Healing,” Photomedicine and Laser Surgery 27, no. 1 (2009): 127–135, 10.1089/pho.2008.2406. 19193104

[33] A. P. C. de Sousa , G. M. Paraguassú , N. T. T. Silveira , et al., “Laser and Led Phototherapies on Angiogenesis,” Lasers in Medical Science 28, no. 3 (2013): 981–987, 10.1007/s10103-012-1187-z. 22923269

[34] M. H. C. de Vasconcelos Catão , C. F. W. Nonaka , R. L. C. de Albuquerque , P. M. Bento , and R. de Oliveira Costa , “Effects of Red Laser, Infrared, Photodynamic Therapy, and Green Led on the Healing Process of Third‐Degree Burns: Clinical and Histological Study in Rats,” Lasers in Medical Science 30, no. 1 (2015): 421–428, 10.1007/s10103-014-1687-0. 25391372

[35] G. I. Klebanov , N. Shuraeva , T. V. Chichuk , A. N. Osipov , and I. Vladimirov , “[A Comparison of the Effects of Laser and Light‐Emitting Diodes on Superoxide Dismutase Activity and Nitric Oxide Production in Rat Wound Fluid],” Biofizika 51, no. 1 (2006): 116–122. 16521561

[36] G. I. Klebanov , N. Y. Shuraeva , T. V. Chichuk , A. N. Osipov , and Y. A. Vladimirov , “A Comparative Study of the Effects of Laser and LED Radiation on Lipid Peroxidation in Rat Wound Fluid,” Biophysics 51 (2006): 285–291.

[37] L. F. de Freitas and M. R. Hamblin , “Proposed Mechanisms of Photobiomodulation or Low‐Level Light Therapy,” IEEE Journal of Selected Topics in Quantum Electronics 22, no. 3 (2016): 348–364, 10.1109/JSTQE.2016.2561201. PMC5215870 28070154

[38] M. T. Pagin , F. A. de Oliveira , R. C. Oliveira , et al., “Laser and Light‐Emitting Diode Effects on Pre‐Osteoblast Growth and Differentiation,” Lasers in Medical Science 29, no. 1 (2014): 55–59, 10.1007/s10103-012-1238-5. 23179312

[39] Y. Y. Huang , A. C. H. Chen , J. D. Carroll , and M. R. Hamblin , “Biphasic Dose Response in Low Level Light Therapy,” Dose‐Response 7, no. 4 (2009): 358–383, 10.2203/dose-response.09-027.Hamblin. 20011653 PMC2790317

[40] N. N. Houreld , R. T. Masha , and H. Abrahamse , “Low‐Intensity Laser Irradiation at 660 nm Stimulates Cytochrome C Oxidase in Stressed Fibroblast Cells,” Lasers in Surgery and Medicine 44, no. 5 (2012): 429–434, 10.1002/lsm.22027. 22488690

[41] S. Farivar , T. Malekshahabi , and R. Shiari , “Biological Effects of Low Level Laser Therapy,” Journal of Lasers in Medical Sciences 5, no. 2 (2014): 58–62. 25653800 PMC4291815

[42] F. Cieplik , A. Späth , C. Leibl , et al., “Blue Light Kills Aggregatibacter Actinomycetemcomitans Due to Its Endogenous Photosensitizers,” Clinical Oral Investigations 18, no. 7 (2014): 1763–1769, 10.1007/s00784-013-1151-8. 24297656

[43] M. S. Wietecha and L. A. DiPietro , “Therapeutic Approaches to the Regulation of Wound Angiogenesis,” Advances in Wound Care 2, no. 3 (2013): 81–86, 10.1089/wound.2011.0348. 24527330 PMC3623575

[44] J. Feng , Y. Zhang , and D. Xing , “Low‐Power Laser Irradiation (LPLI) Promotes VEGF Expression and Vascular Endothelial Cell Proliferation Through the Activation of ERK/Sp1 Pathway,” Cellular Signalling 24, no. 6 (2012): 1116–1125, 10.1016/j.cellsig.2012.01.013. 22326662

[45] V. Cury , A. I. S. Moretti , L. Assis , et al., “Low Level Laser Therapy Increases Angiogenesis in a Model of Ischemic Skin Flap in Rats Mediated by Vegf, HIF‐1α and Mmp‐2,” Journal of Photochemistry and Photobiology, B: Biology 125 (2013): 164–170, 10.1016/j.jphotobiol.2013.06.004. 23831843 PMC3759230

[46] C. C. S. Martignago , R. F. Oliveira , D. A. A. Pires‐Oliveira , et al., “Effect of Low‐Level Laser Therapy on the Gene Expression of Collagen and Vascular Endothelial Growth Factor in a Culture of Fibroblast Cells in Mice,” Lasers in Medical Science 30, no. 1 (2015): 203–208, 10.1007/s10103-014-1644-y. 25171833

[47] S. S. Kuppa , J. Y. Kang , J. Y. Kim , et al., “Red‐Light Led Therapy Promotes Wound Regeneration by Upregulating COL1A1, COL2A1, Vegf and Reducing IL‐1β for Anti‐Inflammation,” Lasers in Medical Science 40, no. 1 (2025): 171, 10.1007/s10103-025-04432-9. 40175683

[48] Q. Sun , H. E. Kim , H. Cho , S. Shi , B. Kim , and O. Kim , “Red Light‐Emitting Diode Irradiation Regulates Oxidative Stress and Inflammation Through SPHK1/NF‐κB Activation in Human Keratinocytes,” Journal of Photochemistry and Photobiology B: Biology 186 (2018): 31–40, 10.1016/j.jphotobiol.2018.05.015. 30005204

[49] F. F. Sperandio , A. Simões , L. Corrêa , et al., “Low‐Level Laser Irradiation Promotes the Proliferation and Maturation of Keratinocytes During Epithelial Wound Repair,” Journal of Biophotonics 8, no. 10 (2015): 795–803, 10.1002/jbio.201400064. 25411997 PMC4583360

[50] S. W. Jere , N. N. Houreld , and H. Abrahamse , “Role of the PI3K/AKT mTOR and GSK3β) Signalling Pathway and Photobiomodulation in Diabetic Wound Healing,” Cytokine & Growth Factor Reviews 50 (2019): 52–59, 10.1016/j.cytogfr.2019.03.001. 30890300

[51] N. C. S. Borges , L. R. Soares , M. M. Perissini , et al., “Photobiomodulation Using Red and Infrared Spectrum Light Emitting‐Diode (LED) for the Healing of Diabetic Foot Ulcers: A Controlled Randomized Clinical Trial,” Lasers in Medical Science 39, no. 1 (2024): 253, 10.1007/s10103-024-04199-5. 39382587

[52] V. Saura Cardoso , P. R. de Souza Lima da Silveira , C. M. Dos Santos , et al., “Dose‐Response and Efficacy of 904 nm Photobiomodulation on Diabetic Foot Ulcers Healing: A Randomized Controlled Trial,” Lasers in Medical Science 39, no. 1 (2024): 142, 10.1007/s10103-024-04090-3. 38805069

[53] E. Austin , A. N. Geisler , J. Nguyen , et al., “Visible Light. Part I: Properties and Cutaneous Effects of Visible Light,” Journal of the American Academy of Dermatology 84, no. 5 (2021): 1219–1231, 10.1016/j.jaad.2021.02.048. 33640508 PMC8887026

[54] M. Cronshaw , S. Parker , M. Grootveld , and E. Lynch , “Photothermal Effects of High‐Energy Photobiomodulation Therapies: An In Vitro Investigation,” Biomedicines 11, no. 6 (2023): 1634, 10.3390/biomedicines11061634. 37371729 PMC10295700

[55] R. G. Frykberg and J. Banks , “Challenges in the Treatment of Chronic Wounds,” Advances in Wound Care 4, no. 9 (2015): 560–582, 10.1089/wound.2015.0635. 26339534 PMC4528992

